# The Pathology of Fevers; or Observations Critical and Experimental in Reference to the Nature, Varieties, Causes and Treatment of Fevers

**Published:** 1855-07

**Authors:** N. S. Davis

**Affiliations:** Prof. of Pathology, Practice, and Clinical Medicine in Rush Med. College; member of the Amer. Med. Association, &c., &c.


ART. VII.—*The Pathology of Fevers; or observations critical and experimental in reference to the nature, varieties, causes and treatment of Fevers.* By N. S. Davis, M. D., Prof. of Pathology, Practice, and Clinical Medicine in Rush Med. College; member of the Amer. Med. Association, &c., &c.

CAUSES OF FEVER.—(*Continued from January Number.*)

If after from five to ten hours fasting, the bulb of a delicately graduated thermometer be inserted under the tongue, with the mouth closed around it for ten minutes, and the same thing repeated an hour after taking a full meal of digestable food, the temperature will be found increased usually to the extent of 1° or 1°5; I have sometimes found it elevated two degrees of Farenheit. It is only a few hours, indeed, since I had an opportunity to put to the test the correctness of this assertion. Having been compelled by professional engagements, to go without food or drink for 18 hours, I found the temperature, indicated by the thermometer, under the tongue to be 97°5, and an hour after dinner, with which a single cup of coffee was drank, the temperature had risen to 99°3, being a fraction less than two degrees.

My experiments on this subject have shown that the temperature of the system increases in proportion to the rapidity of digestion, until that process is nearly completed, when it again begins to decline, and continues gradually diminishing until it reaches the minimum for the healthy individual. This affords a satisfactory explanation of the fact which has long been known, viz:—That the same individual will resist the influence of severe cold much longer when frequently supplied with nutritious food, than when the supply is scanty or entirely cut off. This increase of temperature during digestion may also explain the fact, now pretty well established, that the blood of the vena porte is generally an appreciable degree warmer than either the venous or arterial blood in other parts of the system. Different experimenters have reported different results in regard to the comparative temperature of different internal organs and structures; some claiming that the highest temperature is in the left ventricle of the heart, others in the right, and others again, that it was in the pyloric orifice of the stomach, the mesentery, and the vena-porte.

May not most of these diverse results have been owing to the experiments having been performed at different periods of time after the taking of food?

The facts here developed in reference to the influence of digestion on the *temperature* of the system, fully explain the reason why long fasting or insufficient food so greatly increases the tendency to disease among bodies of men exposed to low temperature, while others exposed to the same degree of cold, but well fed, suffer very little, or none at all. Experience long since demonstrated the fact that in highly malarious districts, those who go out into the fields in the morning *fasting*, are much more liable to an attack of ague, or malarious disease, than those who, though they go out at an hour equally early, uniformly take their breakfast first. The experiments which show that after the long fast of the night the temperature of the body is from one to two degrees lower than after breakfast, afford a satisfactory explanation, inasmuch as the organic actions and vital forces may be regarded as suffering the same depression as the temperature. Another mode by which insufficient nourishment acts as a cause of general fever, is by its alteration of the primary structures, and consequently also of the elementary properties with which these structures are endowed. As all the tissues are constantly undergoing change, the first effect of insufficient supply of nourishment, is to destroy the equilibrium between the nutrition and disintegration. The latter predominating, causes one set of particles to be removed more rapidly than new ones are furnished to fill their places, thereby rendering the very organization of the tissue defective, and lessening what I have styled its tonicity. Again, the same defective supply of nourishment by altering the relative proportion of the nutritive constituents of the blood, would alter in some degree also the mental affinity between the blood and the several structures of the body. This altered affinity or relation of the blood to the primary structures, combines with the impairment of textures, to induce, not the sudden developement of active febrile or other morbid phenomena, but the gradual depression and final failure of the functions of the animal economy. Thus the mind becomes inactive and desponding; the muscular action weak and inefficient; the capillary circulation enfeebled; and the secretions diminished. This state is often seen in what is styled the *forming* stage of Typhoid Fever, especially among immigrants, and the poorer classes in large cities; and may continue from one to three weeks before the more decided febrile phenomena are developed. That defective nutritive material in the blood and consequent alteration of the relative proportion of its constituents is capable of altering the mutual action between the blood and the secretive structures, is plainly shown in the effects of excessive losses of blood. When a considerable quantity of blood is lost, a rapid absorption of water replaces the requisite bulk in the vessels, but leaves the relative proportion of solid constituents diminished for a much longer time. Patients in such a condition often exhibit not merely muscular weakness, coupled with nervous susceptibility, but they exhibit equal impairment of all the secretory processes. We find deficient moisture in the mouth, torpid bowels, scanty urine, dry skin, and sometimes so much inaction in the liver as to allow the coloring matter of bile to render the patient jaundiced over the whole surface; and all our efforts to remove these defects in secretory action fail, until the natural proportion of the nutritive constituents of the blood is restored.

It is not probable that decided febrile action often arises from the *direct* influence of deficient nourishment. But when such deficiency has induced that impairment of tonicity and vital affinity already described, the consequent failure of the secretory functions generally soon result in the accumulation of an excess of effete matter in the system, which, acting upon the previously impaired organic properties, rapidly induce those perverted actions which constitute the common phenomena of the lower grades of fever. In practice, we seldom find deficient nourishment acting *alone*, as a cause of disease. It is almost always associated, either with excessive exercise, defective clothing, or impure air; and often with all these combined.

The effects of prolonged and undue physical exercise, are not very unlike those produced by deficient nourishment. The exercise or natural action of any tissue, is well known to facilitate both the circulation through and the organic changes in the tissue itself. Hence, when such exercise is only regular and moderate it simply favors healthy nutrition; but when too severe or too prolonged it causes the waste of matter from the tissue to predominate over that of supply by nutrition, and the result is an impairment of texture and functions with all the more remote consequences previously described. And it is well known that muscular exercise may be so prolonged as to entirely exhaust the susceptibility of the fibre, and render it incapable of further contraction. The same is true of the involuntary as well as the voluntary system of muscles. Thus, the animal long pursued in the chase, not merely exhausts his voluntary muscles, but sometimes drops dead from the failure of susceptibility and contraction in the heart itself. Soldiers on long and rapid marches, and in the midst of protracted engagements on the battle-field, sometimes die from the same cause. The danger of producing serious consequences from over exercise is greatly increased if accompanied by mental depression, anxiety, or fear.

Much has been written on the *Mind* and its alleged mysterious connection with the body, and yet the influences of its various conditions in favoring or retarding the development and progress of disease is but imperfectly appreciated. All know that, as a general rule, a cheerful, placid, and hopeful condition of mind gives rise to a free circulation, active innervation, and healthy nutrition; effects which tend strongly to counteract the development of morbid action in any part of the animal economy. Hence, individuals enjoying habitually these qualities of mind, are both less liable to attacks of disease, and more likely to recover when attacked, than almost any other class. For the same reason they are apt to live much beyond the average duration of human life. While such are the happy influences of cheerfulness and hope on the physical system, excessive hilarity habitually indulged favors nervous excitability, and joy sometimes excites the heart to inordinate contractions, inducing dangerous congestion if not sudden death. On the other hand, despondency, grief, and fear, directly depress or lessen all the elementary properties and functions of the physical system. Under their influence the heart acts feebly, the capillary circulation is less active, respiration is slower and less full, while the whole muscular system is relaxed and feeble. When these mental conditions are strongly marked and long continued, they may so far impair the various functions of the body, as to develop a form of slow nervous or typhoid fever, without the intervention of any other cause. And they at all times greatly increase the predisposition or susceptibility to disease. Thus it is well known that in every season of pestilence, the timid, the desponding, and anxious ones, are far more liable to an attack than those of an opposite mental temperament; and for the same reason they are less likely to recover under the use of the same remedial agents.

These influences of the mind over the body are no more mysterious than any of the other phenomena of life. The brain and nerve tissue, like all other living organized matter, is endowed with its own susceptibility, and the emotions and passions of the mind, as well as the outward objects acting through the senses, are so many excitors or agents capable of exerting as direct an influence on the properties of the brain, as the air does on the lungs or the blood on the heart. Hence, pleasant or exhilerating emotions and impressions increase the susceptibility and tone of the brain or nerve structure, in the same manner that well oxygenated blood increases the susceptibility and contractility of the muscular structures, both voluntary and involuntary. And impure or deficiently oxygenated blood does not more certainly fail to maintain the normal condition of the elementary properties of the muscular structures, than do the saddening emotions and impressions of the mind to preserve in full force the same properties of the brain.—If this be true, and we keep in mind the physiological relations of the brain and nervous centers to all the movements which take place in the system, whether voluntary or involuntary, we shall find no difficulty in conceiving why an emotion or sensation which directly depresses the elementary properties of the brain, should also relax the sphincters, render the muscular system feeble and agitated, and diminish the action of the organs both of circulation and respiration. A knowledge of this subject is of much importance to the practicing physician; for when rightly understood, the various mental conditions furnish as valuable prophylactic and remedial agents as any of the articles of the Materia Medica.

*Impure Air.*—The term, impure air, so generally urged as a cause of febrile disease, is very indefinite. In its general sense, it includes all conditions of the atmosphere, characterized by the addition of anything to its natural constituents. It is more commonly, restricted,however,to those conditions of the atmosphere produced by the diffusion in it of the excrementitious matters emanating from the living animal body, and the various products arising from the decomposition of the refuse vegetable and animal matters which always accumulate more or less in cities, and all places where many persons are congregated on a small space of ground. The first class of impurities, namely, those arising directly from the living body, become efficient and active as causes of fever chiefly when concentrated in dwellings and lodging rooms, and are consequently derived mostly from the lungs and skin.

The first, as is well known, eliminates from eight to twelve ounces of carbon in the form of corbonic acid gas, every twenty-four hours, and the latter from twenty-five to thirty ounces during the same period of time; making the aggregate daily elimination from the skin and lungs of a healthy adult more than 40 oz. per day. Nearly all this matter escapes in the form of gasses and aqueous vapor. It consists chiefly of carbonic acid gas, aqueous vapor, and saline matter; but in all cases there is intermixed a sufficient proportion of *animal* matter to be capable of undergoing decomposition or putrefaction. According to the analysis of Mr. Ancell, of London, the amount of *animal* matter contained in the elimination from the skin and lungs of a healthy adult during 24 hours, averages about half an ounce. Nearly all of this is connected with the aqueous vapor and saline matter which escapes through the cutaneous surface. Taking these data as the basis of estimates, it is not difficult to compute the impurity which would be communicated to any limited amount of atmospheric air by a given number of persons. Dr. Joseph M. Smith, of New-York, in an interesting report made to the American Medical Association, in May, 1850, computed the amount thrown off by a family of ten adult persons, within their own dwelling, as follows, viz:

"Let us suppose a family, of which there are hundreds of examples, consisting of ten adult persons, dwelling in a small, ill-ventilated house, and negligent of personal or domestic cleanliness; and further, that the time severally passed within doors by the ten individuals, some of whom are constantly at home, while others are temporarily absent, amount in the aggregate to twelve hours out of every twenty-four. The mass of effete matter thrown out by the lungs and skin, by such a family within their dwelling, in one month, is 500 lbs.; in six months, 3033 lbs. 4 oz.; and in one year, 6083 lbs. 4 oz. Though by far the greater part of these excretions consist of carbonic acid, water, and salts, yet the quantity of ejected *animal* matter is not inconsiderable. It amounts in one month to 6 lbs. 3 oz.; in six months to 37 lbs. 11 oz.; and in one year to 76 lbs."

Again the same writer says:—"Instead of a hypothetical example, let us select one which is reported as actually existing.—'In one of the *cul-de-sacs* in the town of Leeds, there are thirty-four houses, (described as impure and ill-ventilated,) and in ordinary times there dwell in these houses 340 persons, or ten to every house. It is probable that of the 340 persons who ordinarily occupy the thirty-four houses some are children; yet, though this may be true, the occasional itinerant lodgers doubtless make the number residing in each house throughout the year equivalent to ten adult persons. Now if the cutaneous and pulmonary excretions of one adult be 40 oz. per day, then the 340 adults discharge from the lungs and skin within their dwellings, in the half of one day, or twelve hours, (assuming that this is the average of the time passed severally by the whole number in their houses,) 566 lbs, 8 oz, and in the same proportions, in one month 17,000 lbs.; in six months, 103,133 lbs. 4 oz.; and in one year, 206,833 lbs. 4 oz. It is said there were rémoved from the place in which the houses in question are situated, during the days of cholera, 75 cart loads of manure—a quantity, allowing each cart load to weigh 1200 lbs., not equal to half of the amount of materials eliminated from the skin and lungs of the 340 persons, within the thirty-four houses, in one year.

If we now calculate the amount of *animal* matter cast out from the 340 persons, in the half of each day, it will be found to be 7

lbs. 1 oz. ; and, in corresponding proportions for each day, in one month, 212 lbs. 5 oz. ; in six months, 1289 lbs, 6 oz. ; and in one year, 2585 lbs. 5 oz. ; this last amount being more than two cart loads, each of the weight above mentioned.' "

The reader will easily perceive by these estimates, founded as they are on many carefully performed experiments, that the effete matters passed off in the form of fœces and urine, constitute really but a minor part of what is daily discharged from the system. It is true that of the large quantity thrown out from the skin and lungs, the greater part is in that gaseous or æriform state which causes it to become rapidly diffused, in the atmosphere, and too much diluted to produce evil consequences. Still it will be fully apparent, that when houses are over crowded, or situated in narrow streets, with obstructions to the free circulation of air, or when lodging rooms are small and imperfectly ventilated, even the more diffusable gaseous exhalations, such as the carbonic acid, are accumulated to a degree highly deleterious to health. But the animal matter, the greater part of which is eliminated in connection with the aqeous vapor from the skin, is much less likely to undergo rapid or complete diffusion in the air. Some of it is always retained in the clothing which covers the body, and much of that which actually becomes diffused in the dwellings and lodging rooms along with the vapor, is condensed and becomes attached to the furniture and walls of the apartments, where it may remain until decomposed or chemically changed. It is this effete animal matter, more than any other excrementitious matter of the system, which, if not positively poisonous when eliminated, is always liable, when suffered to accumulate, to undergo further changes, by which those virulent animal poisons are generated that give rise to the more malignant forms of typhoid fever, dysentery, and erysipelas.

And it is only on the supposition that these poisons, when once generated, are capable of becoming attached to the walls of apartments, and furniture, that we can account for the fact sometimes observed of the occurrence of the same disease in the successive tenants of particular rooms, while others perhaps in the same building are entirely exempt. Many examples of this kind are on record ; some in which rooms made entirely vacant during several months after typhus fever had occured in them, yet when re-occupied, the same disease again attacked the inmates.

